# Pregnancy associated anti-TIF1 dermatomyositis responsive to intravenous immunoglobulin

**DOI:** 10.1016/j.jdcr.2023.11.006

**Published:** 2023-11-30

**Authors:** Jason Kieffer, Chenan Andy Huang, William Steffes

**Affiliations:** a Department of Dermatology, KCU-GME/ADCS Consortium, Maitland, Florida; b University of Central Florida College of Medicine, Orlando, Florida

**Keywords:** dermatomyositis, intravenous immunoglobulin, IVIG, myositis, photosensitive, pregnancy, TIF1

## Introduction

We present a case of new onset dermatomyositis (DM) in a patient during the second trimester of pregnancy. With an estimated 1 to 6 new cases per 100,000 in the US annually and an average age of diagnosis occurring after child-bearing age (40 to 60-years-old),^1^ this case highlights a unique clinical challenge in managing DM while optimizing maternal and fetal outcomes.

## Case report

**Fig 1:**
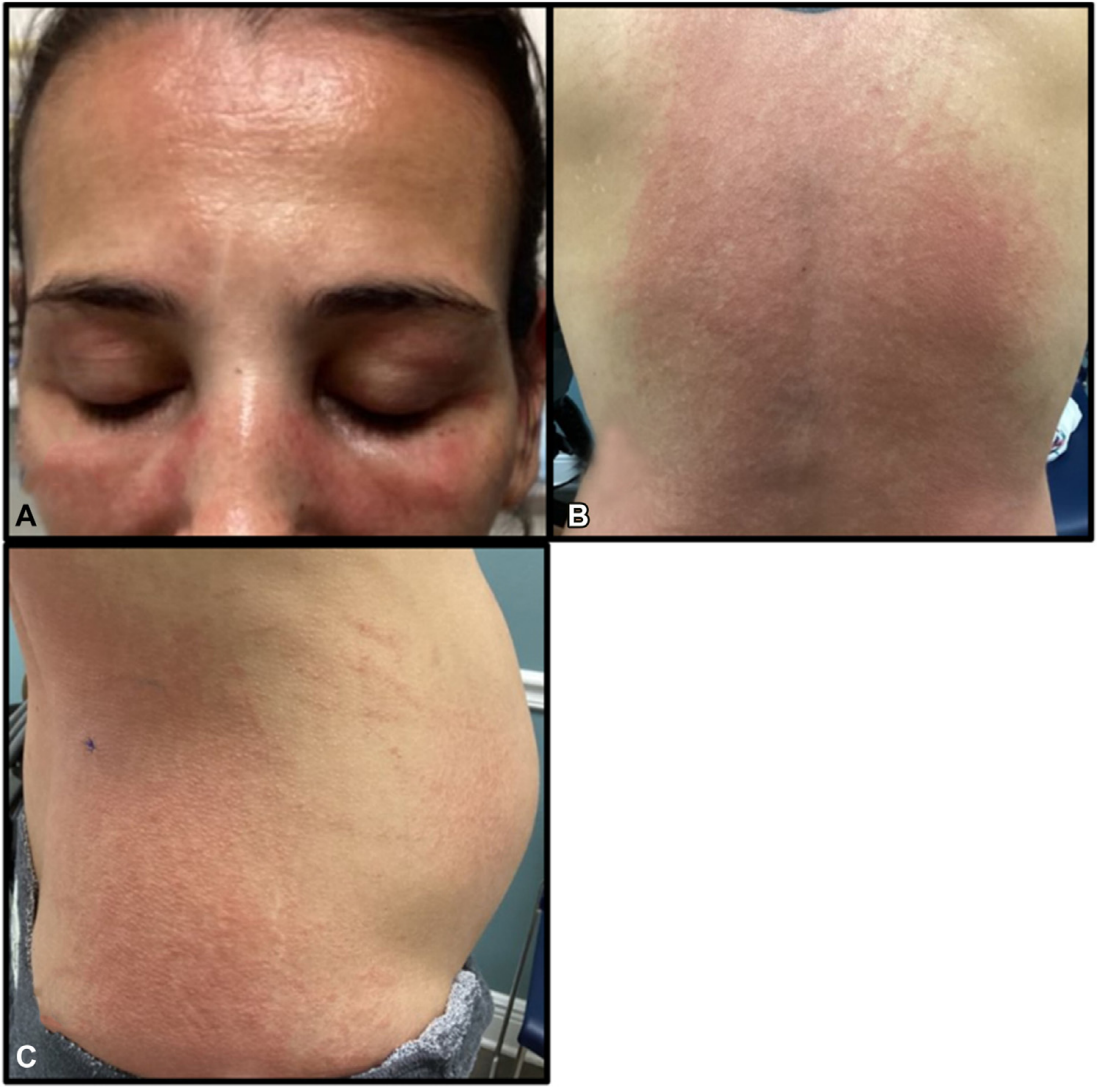
Edematous heliotrope rash on malar cheeks, eyelids, and forehead (**A**). Erythematous papules and plaques with flagellate morphology on back (**B**) and anterolateral trunk with gravid abdomen (**C**).

**Fig 2:**
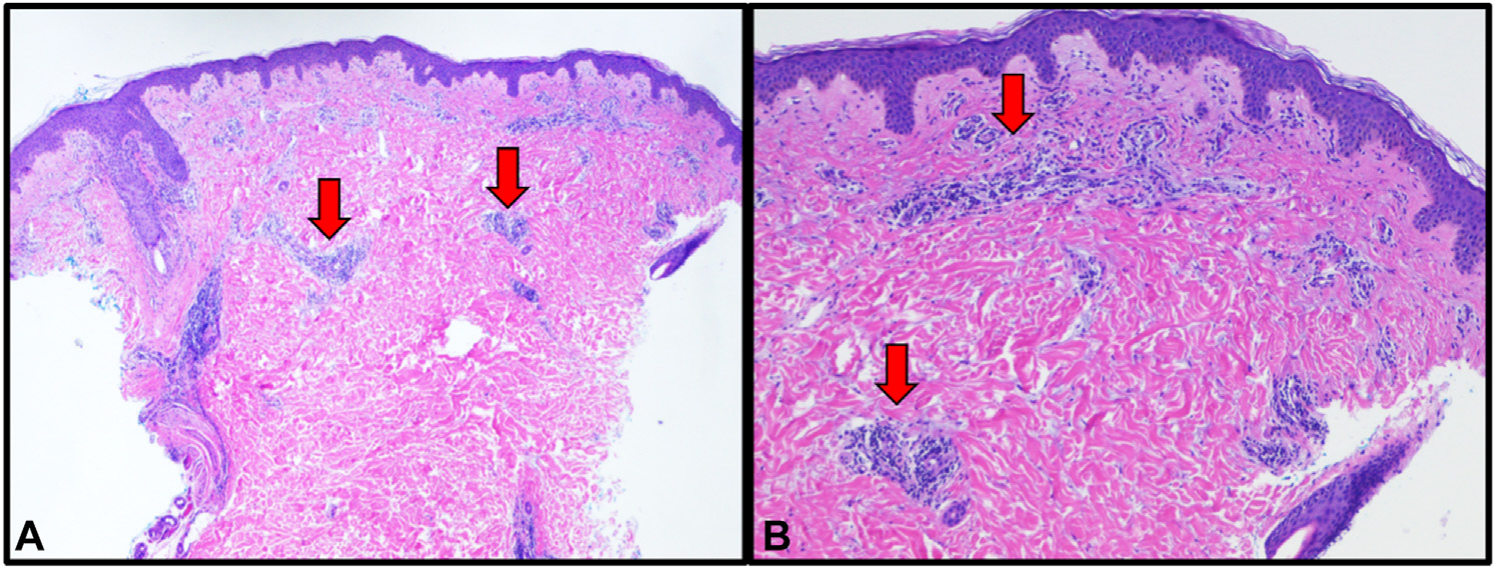
Punch biopsy demonstrating dermal perivascular lymphocytic infiltrate consistent with dermatomyositis. H&E 40× (**A**), 100× (**B**) magnification. *H&E*, Hematoxylin and eosin.

A 31-year-old G2P1 woman at 24 weeks gestation initially presented with a mild erythematous rash of the malar cheek and superior chest. The rash was exacerbated following sun exposure from a beach outing, and spread to her trunk, lateral arms, thighs, and dorsal hands. The eruption was composed of erythematous papules coalescing into plaques and featured flagellate morphology at the periphery of truncal lesions (Fig 1). Additionally, her face exhibited an edematous heliotrope rash (Fig 1). She reported associated symptoms of fatigue, mild dysphagia, and occasional dyspnea. Prior medical history was insignificant, with 1 uncomplicated term pregnancy and no medications other than prenatal vitamins. Serum studies were notable for elevated CK 1008 u/L (24-200 u/L), aldolase 8.9 u/L (1.2-7.6 u/L), and anti-TIF1 antibody positivity. H&E evaluation of punch biopsies from the inferolateral back and lateral thigh both revealed a perivascular lymphocytic inflammation supporting a diagnosis of DM (Fig 2). Initial treatment with prednisone 60 mg daily stabilized her condition and improved her dysphagia and fatigue but had minimal effect on her cutaneous findings. She was referred to perineonatology, gastroenterology, and oncology for malignancy screening. Topical steroids and oral hydroxychloroquine were started as adjunctive therapy with little improvement. While attempting to taper prednisone, her condition flared at 40 mg requiring an increase to 70 mg daily. After coordination with her obstetrician, we decided to start IVIG.

At 32 weeks gestational age, she received her first infusion of IVIG dosed at 2 g/kg and noted significant improvement in her symptoms within days. Symptoms remained stable as the prednisone dose was titrated downward to 40 mg daily. A second infusion of IVIG was given a month later at 36 weeks gestation and her prednisone dose was decreased to 20 mg daily over the next 2 weeks. The patient delivered a healthy neonate at term and reported complete resolution of symptoms within a week following delivery. Blood work performed at 1-week post-partum confirmed normalization of CK levels at 40 u/L. Evaluation for underlying malignancy associated with anti-TIF1 DM revealed normal serum CEA, CA19-9, and CA-125 as well as normal esophagogastroduodenoscopy, colonoscopy, and CT imaging of the chest, head, and neck. CT of the abdomen and pelvis was deferred to avoid radiation to the fetus. The patient moved out of the country and was lost to follow up at 5 weeks postpartum, at which time she remained asymptomatic while continuing to taper from prednisone 10 mg daily.

## Discussion

Although anti-TIF1 antibody DM is correlated with malignancy, in patients of childbearing age, the trigger may be high TIF1 expression in both fetal tissues and lactating mammary epithelium.^2^ A case series by Oya et al. identified 7 patients of childbearing age (15-49) who developed anti-TIF1 positive DM, 3 of whom developed DM during pregnancy and the postpartum period.^2^ Only 1 patient developed DM related to malignancy. In contrast, 10 of 16 patients with anti-TIF1 positive DM and of non-childbearing potential were related to malignancy. Fetal microchimerism in maternal skin provides a route of sensitization and disease progression. In paraneoplastic anti-TIF1 DM, resection of tumors revealed high TIF1 expression and resulted in clinical improvement. These data suggest that sensitization to TIF1 antigens, whether in fetal antigens or in malignant cells, may be a mechanism underlying anti-TIF1 positive DM.^2^ Despite this proposed mechanism, a definitive link has not been established. Other myositis autoantibodies have been recorded to occur in gravid and post-partum cases of DM,^3^ suggesting the state of pregnancy itself may influence disease onset. While further studies are needed to validate the pathophysiologic mechanism, our case fits within the framework described by Oya et al. and indicates the potential for remission after reduction of TIF1 antigens, similar to improvement after tumor resection in paraneoplastic DM.

Tang et al.^4^ sought to summarize the current literature on pregnancy outcomes in DM and polymyositis (PM). Their systematic review identified 61 studies, the majority consisting of case reports and case series. Across 221 pregnancies, they demonstrated that active disease resulted in significant risks of stillbirth, preterm birth, and other poor fetal outcomes (ie low birth weight).^4^ The retrospective cohort by Kolstad et al. reviewed 853 deliveries from patients with DM or PM and evaluated maternal outcomes. Their analysis indicated longer hospitalizations and increased risk of hypertensive disorders in these patients, but no difference in rate of cesarean section.^5^

Limiting the extent of active disease, therefore, seems vital to both maternal and fetal health. Systemic steroids alone are not always sufficient to induce remission, and at prolonged high dosages, come with risk to mother and fetus. Adjunctive treatment options for our patient were considered following the recently published therapeutic algorithm on DM.^1^ Several common treatments utilized in DM are either absolutely contraindicated in pregnancy (methotrexate, mycophenolate mofetil, and rituximab)^6^, ^7^, ^8^ or carry significant comorbid risk (cyclosporine).^9^ Hydroxychloroquine may be used during pregnancy but has limited benefits on the systemic symptoms of DM.^1^ Meanwhile, IVIG is considered a safe and effective therapy in several maternal conditions.^10^ The systematic review by Tang et al. identified 8 publications supporting IVIG in pregnancy related cases.^4^

Reduction of anti-TIF1 antigens and their inflammatory effects via immunomodulation or parturition appears effective from both Oya et al and our experience in this case. Immunomodulation via steroids was effective as an initial stabilizing treatment, with additional efficacy achieved through IVIG therapy. Meanwhile, removal of anti-TIF1 antigens via parturition is possibly the mechanism accounting for our patient’s postpartum remission. We believe this case is notable as it further illustrates the excellent capability of IVIG to safely mitigate the maternal and fetal risks associated with DM to ensure successful pregnancy outcomes.

## Conflict of interest

None disclosed.
